# Height of overburden fracture based on key strata theory in longwall face

**DOI:** 10.1371/journal.pone.0228264

**Published:** 2020-01-24

**Authors:** Weiyong Lu, Changchun He, Xin Zhang

**Affiliations:** 1 Department of Mining Engineering, Luliang University, Lvliang, Shanxi, P.R. China; 2 School of Civil Engineering and Architecture, East China University of Technology, Nanchang, Jiangxi, P.R. China; 3 School of Minerals and Energy Resources Engineering, Faculty of Engineering, University of New South Wales, Sydney NSW, Australia; Sapienza Universita di Roma, ITALY

## Abstract

Among the three overburden zones (the caving zone, the fracture zone, and the continuous deformation zone) in longwall coal mining, the continuous deformation zone is often considered to be continuous without cracks, so continuum mechanics can be used to calculate the subsidence of overburden strata. Longwall coal mining, however, will induce the generation of wide cracks in the surface and thus may cause the continuous deformation zone to fracture. In this paper, whether there are cracks in the continuous deformation zone as well as the height of overburden fracture in longwall face and the subsidence and deformation of strata of different fracture penetration ratios were studied by means of physical simulation, theoretical analysis and numerical simulation. The results show that: (1) Rock stratum starts to fracture as long as it has slightly subsided for only tens of millimeters, and the height of fracture development is the height of working face overburden. (2) With the increase of fracture penetration ratio, the subsidence of key strata remains basically unchanged; the surface deformation range and the maximum compression deformation decrease, while the maximum horizontal movement and maximum horizontal tensile deformation increase. Therefore, the subsidence of overburden strata which have fractured but have not broken can be calculated through the continuum mechanics method.

## Introduction

Coal mining will lead to roof caving, overburden fracturing, surface subsidence and environmental damage, resulting in groundwater pollution, surface water flow cutoff, vegetation withering and farmland waterlogging [1–10]. Therefore, it is especially important to study the surface subsidence caused by coal mining. Field observations and physical model simulations have shown that the key strata (KS) dynamically controls the surface subsidence [11–12], which means that the KS and the controlled soft rocks obtain synchronous subsidence and subsidence speed. This suggests that in order to accurately predict surface subsidence, it is necessary to first calculate KS subsidence within overburden. As a result, many scholars began to use continuum mechanics to calculate KS subsidence within overburden [8,13,14]. The use of continuum mechanics was based on the traditional knowledge that no fracture exists in strata of continuous deformation zone [15, 16]. However, after longwall mining, the surface subsidence coefficient gets greater than 0.6 which is relatively large for medium-thick coal seams [17], so there will usually be some wide fractures in the surface of these coal seams. This indicates that fractures may also exist in strata of continuous deformation zone, and it is doubtful whether KS subsidence can be calculated by using the continuum mechanics method.

The strata with different distances from the coal seam show different deformation characteristics after the coal seam mining. According to the needs of mining engineering, stabilized overburden strata after movement are roughly divided into three zones, namely, the caving zone, the broken zone and the continuous deformation zone [15, 16, 18–20]. As the caving zone and the broken zone can both conduct water, they are referred to as the water-conducting fractured zone. Mining overburden generally contains several aquifers, and meanwhile water inrush can be very harmful to the stope [21, 22], so mining engineers and scholars have attached great importance to the study of the height of water-conducting fractured zone [17, 21–24]. Different methods are adopted in different countries to calculate the height of water-conducting fractured zone. In China, there are mainly two methods for calculating this height as shown in Table 1, one of which is the empirical formula of statistical data for many years (prescribed method I) and the other is the determination method based on the position of overburden KS (prescribed method II) [17, 23]. Traditionally, it is considered that there is no fracture in the continuous deformation zone. In fact, the fracturing state of this zone is rarely reported, because its relationship with stope is weak and its relationship with surface is just discovered in recent years [11–13].

**Table 1:** Height of broken zone and the mining thickness (M).

Types	Strength	Height of the fractured zone/m
Method I	Strong	$\frac{100\sum M}{1.2\sum M+2.0}\pm 8.9$
	Medium strong	$\frac{100\sum M}{1.6\sum M+3.6}\pm 5.6$
	Weak	$\frac{100\sum M}{3.1\sum M+5.0}\pm 4.0$
	Very weak	$\frac{100\sum M}{5.0\sum M+8.0}\pm 3.0$
Method II		Height of the overburden strata controlled by the KS closest to (7–10) M when the KS is within (7–10) M
		Height of the overburden when the PKS is out of (7–10) M

Continuum mechanics is widely used in coal mines with complex geological conditions, including the calculation of weighting step, major influence radius, strata subsidence, etc. [25–29]. However, studies have shown that the subsidence of strata fully penetrated by joints differs from that of intact strata [30, 31], which shows that continuum mechanics is not suitable for use in the broken zone. Because of this, Academician Qian proposed a mechanical model based on voussoir beam theory [32] which calculated KS movement in the broken zone using the block theory. Although the block theory [33] is well developed, it is not applicable to the calculation of subsidence in the continuous deformation zone that has not been completely penetrated by fractures. If continuum mechanics can be used to calculate this kind of subsidence, the solving process will be greatly simplified, and the engineering application can be made possible as well.

In this paper, the development of fractures in the overburden of continuous deformation zone was qualitatively analyzed firstly by means of physical simulation, and then the critical subsidence conditions of strata fracture were given by combining mechanics theory. Finally, the correctness of theoretical analysis was qualitatively verified by means of numerical simulation, and deformation and movement of strata of different fracture penetration ratios were studied. For KS theory, it was defined that the strength and hardness of KS in the continuous deformation zone are both greater than those of other rock strata, and the movement of KS controls the movement of soft rock above them [15]. Therefore, the study of fracture characteristics of KS in the continuous deformation zone can represent fracture characteristics of the entire continuous deformation zone.

## Physical simulation of fracture development in the continuous deformation zone

### Experimental scheme

In coal mining engineering, two-dimensional model is often used to simulate the fractures and subsidence of longwall overburden strata, and it has the following advantages compared with three-dimensional model: (1) It is simple to make 2D models and can simulate the experimental results in a short time; (2) The simulation results are easy to monitor, and we can intuitively see the fractures of overburden strata, which has a strong convincing force for the confirmation of the conclusion, but 3D simulation is difficult to see the fracture development of overburden strata. In view of this, the 2D model was taken in this paper.

**Fig 1:**
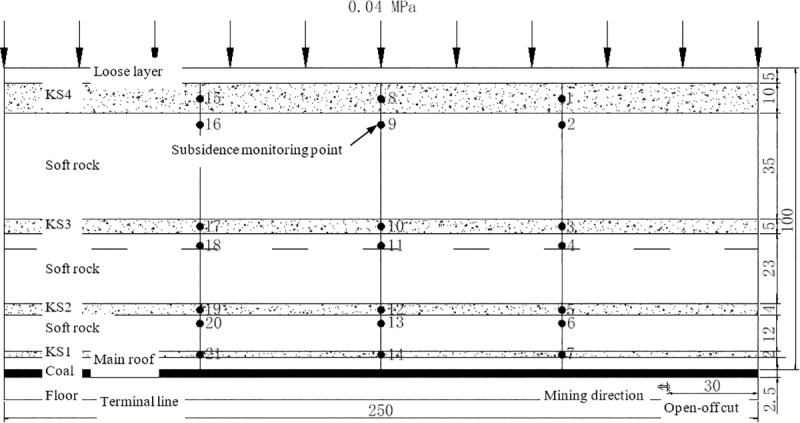
Conceptual diagram of fracture development in physical simulation.

In the simulation experiment, a 2.5 m×0.2 m×2.0 m plane stress model frame was employed. Fig 1 presents the actual size of the model with the geometrical similarity ratio *C*_*L*_ = 1:100, the bulk density similarity ratio *C*_*γ*_ = 1:1.6 and the stress similarity ratio *C*_*σ*_ = 1:160. According to the similarity theory, the physical and mechanical parameters of each stratum were determined and the material ratio was calculated. As for physical simulation materials, river sand and mica were taken as the aggregate, while calcium carbonate and gypsum were taken as the binder. The mining height of the coal seam is 4 m. The KS1, KS2, KS3 and PKS is 4 m, 18 m, 45 m and 85 m above the coal seam with a thickness of 2 m, 4 m, 5 m and 10 m, respectively. The immediate roof is 4 m thick. The relative thickness of the soft rock between KS1, KS2, KS3 and PKS was 12 m, 23m and 35m, respectively. At the top of the model, 0.04 MPa of uniformly distributed load is applied, which is equivalent to the load of 32 m loose layer.

A total of 21 subsidence monitoring points are arranged on the model, among which 12 are arranged in the KS, and 9 are arranged in the soft rock. Monitoring point #8 is arranged in the PKS with equal distance from the left and right sides. The position of subsidence monitoring points is shown in Fig 1.

### Experimental results and analysis

**Fig 2:**
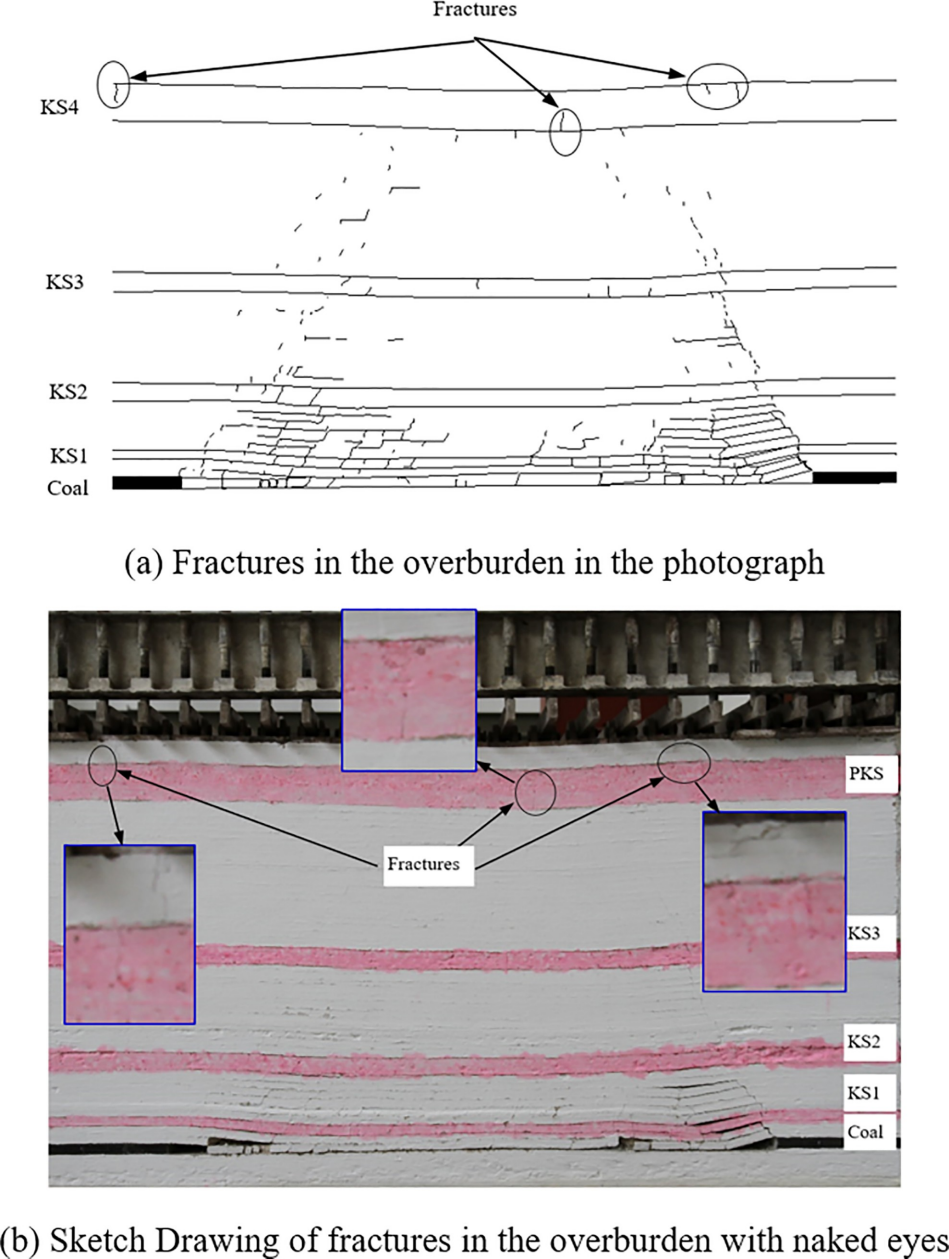
Fractures in the overburden of physical simulation.

**Fig 3:**
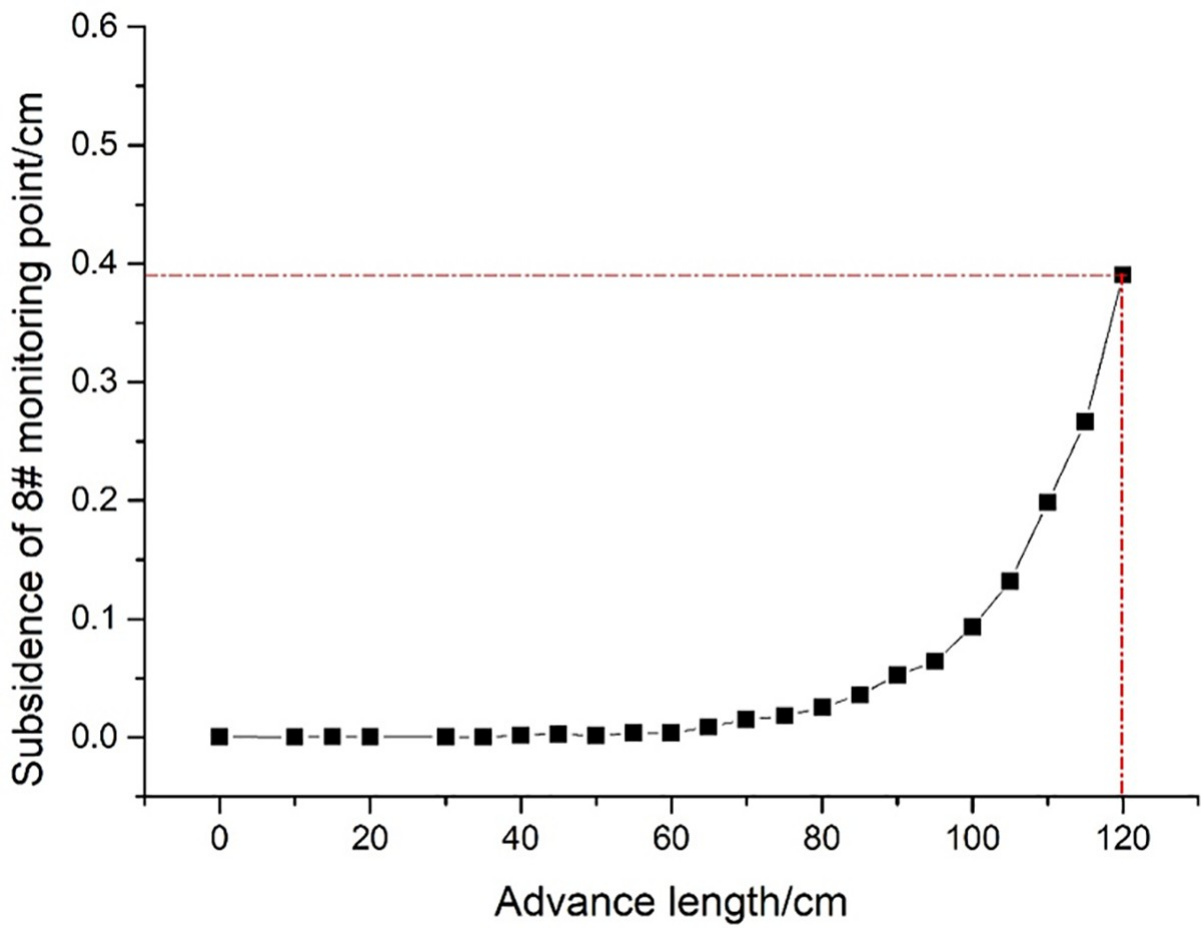
Subsidence of No. 8 monitoring point with advance length.

In the excavation process, boundary protective coal pillars of a 30 cm width were reserved on both sides of the model. The distance for each excavation of the coal seam was 5 cm, corresponding to an actual advance distance of 5 m each time. Fig 2 presents the development of mining-induced fractures in the overburden after 120 m of excavation. According to the whole formulae of the height of water-conducting fractured zone (see Table 1), it can be obtained that KS3 and the strata above it are all within the range of continuous deformation zone. As can be clearly seen from Fig 2, fractures exist in all strata within the continuous deformation zone. That is, the critical conditions of strata fracturing have been reached. The relationship between subsidence and advance distance at No. 8 monitoring point is shown in Fig 3. The maximum subsidence of stabilized KS4 is about 4 cm after the working face is excavated for 120 cm, yet the KS4 has already fractured before the working face advances to 120 cm.

The immediate roof start to collapse when the coal face advances 40 cm. And at the advance distance of 50 cm, 70 cm and 90 cm, the KS1, KS2 and KS3 start to break down respectively. When the advance distance is 110 cm, fractures in the PKS can be seen with naked eyes, but the fractures do not break through the PKS. Until the end of model excavation, the fractures still do not break through the PKS.

## Critical conditions of rock fracture

**Fig 4:**
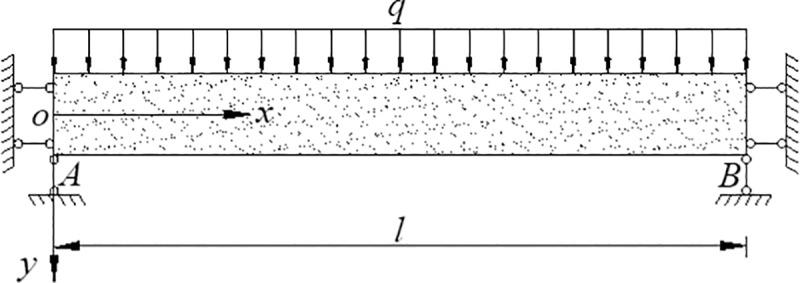
Two ends fixed beam under uniformly distributed loading.

Stratification is a common feature of sedimentary rocks, volcanic rocks and metamorphic rocks, and horizontal stratigraphic rock mass resembles a flat slab. Thus, the plate model can be used to study KS movement and deformation [34]. According to the characteristics of underground coal mining and the subsidence focus of mining area, the subsidence of longitudinal and transverse main sections can represent the whole subsidence range [35–38], the KS of continuous deformation zone can be simplified as a plane strain beam whose stress analysis is of no essential difference from that of plane stress beam [39], so this paper assumed that the overburden of continuous deformation zone was a plane stress beam model. A certain KS of continuous deformation zone was assumed to be a beam fixed at both ends, as shown in Fig 4. The width of the beam was taken as one unit, and the height and length were *h* and *l*, respectively.

As a kind of brittle material, the rock may generate cracks when the stress on a certain point exceeds its strength limit. Hence, to study the crack initiation of KS, it is necessary to focus on whether the maximum stress has exceeded the limit value. Under the uniformly distributed lateral loading, the stress and the subsidence of each point in the KS in the elastic stage) are [40]:

$$\begin{array}{l} \sigma_{x}=q\left[4y^{3}+6\left(l-x\right)xy-\left(l^{2}+h^{2}-\mu h^{2}\right)y-\mu h^{3}/2\right]/h^{3}\\ \sigma_{y}=-q\left(4y^{3}-3h^{2}y+h^{3}\right)/\left(2h^{3}\right)\\ \tau_{xy}=3q\left(2x-l\right)\left(4y^{2}-h^{2}\right)/\left(4h^{3}\right)\\ v=q\left\{\begin{array}{l} -2\left(1+2\mu \right)y^{4}+\left(-2h^{2}\mu^{2}+2h^{2}\mu +2l^{2}\mu -12l\mu x+12\mu x^{2}+3h^{2}\right)y^{2}+\\ 2\left(\mu^{2}-1\right)h^{3}y+5h^{2}l\mu x-5h^{2}\mu x^{2}+4h^{2}lx-4h^{2}x^{2}+2l^{2}x^{2}-4lx^{3}+2x^{4} \end{array}\right\}/\left(4Eh^{3}\right) \end{array}$$ (1)

where *σ*_*x*_, *σ*_*y*_ and *τ*_*x*_ are the normal stress and shear stress respectively. *l* and *h* are the length height of the beam. *μ* is the Poisson's ratio. *q* is the uniformly distributed stress.

According to the actual situation and the physical simulation experiment, it can be known that the crack initiation always takes place first at the upper surface of two fixed ends and at the lower surface of middle part, and the stress at the two positions is:

$$\begin{array}{l} \sigma_{x|x=0/l,y=-\frac{h}{2}}=\frac{q\left(l^{2}-2\mu h^{2}\right)}{2h^{2}},\;\sigma_{y|x=0/l,y=-\frac{h}{2}}=q,\;\tau_{xy|x=0/l,y=-\frac{h}{2}}=0\\ \sigma_{x|x=l/2,y=\frac{h}{2}}=\frac{ql^{2}}{4h^{2}},\;\sigma_{y|x=l/2,y=\frac{h}{2}}=\tau_{xy|x=l/2,y=\frac{h}{2}}=0 \end{array}$$ (2)

The first strength theory demonstrates that for brittle materials, tensile failure is the main reason for the occurrence of cracks. Therefore, the first strength theory should be regarded as the criterion for crack initiation of KS. The strata start to crack when the maximum tensile stress reaches the tensile strength limit *σ*_b_. The Poisson’s ratio of hard rock is small, and its thickness is smaller than the breaking length, so the term containing Poisson’s ratio is negligible. As can be seen from Eq (2), tensile stress at the end is greater than that at the middle part, indicating that crack initiation first takes place at the upper surface of the two fixed ends and then at the lower surface of the middle part. Therefore, tensile stress at the end should be taken to judge whether cracks have been generated. Factors affecting the maximum tensile stress of rock strata include lateral load, suspended length and rock strata thickness. The lateral load is related not only to the overburden structure, i.e., the full columnar of overburden, but also to the breakage characteristics of soft strata controlled by KS. Whether the soft strata controlled by KS has broken at the moment of crack initiation of KS has a great influence on the calculation of load on KS. However, as long as the load on KS calculated by the composite beam theory can cause crack initiation of strata, KS must have cracked when the rock strata controlled by them break. Hence, the load on KS in the continuous deformation zone can be calculated using Eq (3) [15]:

$$q=\frac{E_{1}h_{1}^{3}(\sum_{1}^{n}\gamma_{i}h_{i})}{\sum_{1}^{n}E_{i}h_{i}^{3}}$$ (3)

where, starting from the calculated KS, strata were numbered from bottom to top in an ascending order; *E*_*i*_ is the elastic modulus of KS and strata controlled by KS; and *h*_*i*_ is the thickness of KS and strata controlled by KS.

When the maximum stress at both ends of KS satisfies Eq (4), KS starts to undergo tension failure at the upper surface of the two ends fixed beam. Nevertheless, the fracture will not further develop if the load on KS does not increase.

$$\sigma_{\max}=\frac{E_{1}h_{1}(\sum_{1}^{n}\gamma_{i}h_{i})l^{2}}{2\sum_{1}^{n}E_{i}h_{i}^{3}}=\sigma_{b}$$ (4)

While the actual stress is often hard to measure, the subsidence of KS is an indicator easy for observation. Therefore, it is necessary to derive the maximum subsidence at the moment of KS fracture. For a beam fixed at both ends, the maximum subsidence occurs in the middle part of the beam. The subsidence in Eq (1) can be directly used only when KS stay in the elastic stage. According to the rock mechanics test, before the rock stress reaches the tensile strength, the overall curve is a concave curve that satisfies the polynomial function. That is, the rock does not satisfy the Hooke’s law. However, to simplify the calculation, it is assumed that the stress-strain function can still be expressed by a linear function. Such simplification has little effect on the calculation results of subsidence. This is because (1) the stress-strain curve of the hard rock before failure is very close to a straight line [41]; (2) this paper focuses on the subsidence of macro-cracks, which is independent of the non-linear stress-strain relationship before the fracturing point. After the simplification, the maximum subsidence at both ends of the fixed beam is:

$$v_{\max}=\frac{ql^{2}\left(l^{2}+8h^{2}\right)}{32Eh^{3}}$$ (5)

In addition, the block length has a certain relationship with the load and the tensile strength when KS in the continuous deformation zone break. Eq (6) can be obtained according to material mechanics:

$$l=h\sqrt{\frac{2\sigma_{b}}{q}}$$ (6)

Through combining Eqs (1), (2), (5) and (6), the maximum subsidence critical value at the moment of crack initiation at both ends of the fixed beam can be obtained as follows:

$$v_{\lim}=\sigma_{b}\left(\frac{2\sigma_{b}\sum_{1}^{n}E_{i}h_{i}^{3}}{Eh\sum_{1}^{n}\gamma_{i}h_{i}}+8h^{2}\right)/\left(16Eh\right)$$ (7)

For a given full columnar, the limit subsidence value at the moment of crack initiation of KS can be obtained, as long as the lithology and thickness of KS itself and the elastic modulus and thickness of soft rock controlled by KS are known. If the actual measured subsidence value is greater than the limit subsidence value, it can be judged that the crack has occurred in KS and the soft rock controlled by KS has also been cracked.

**Fig 5:**
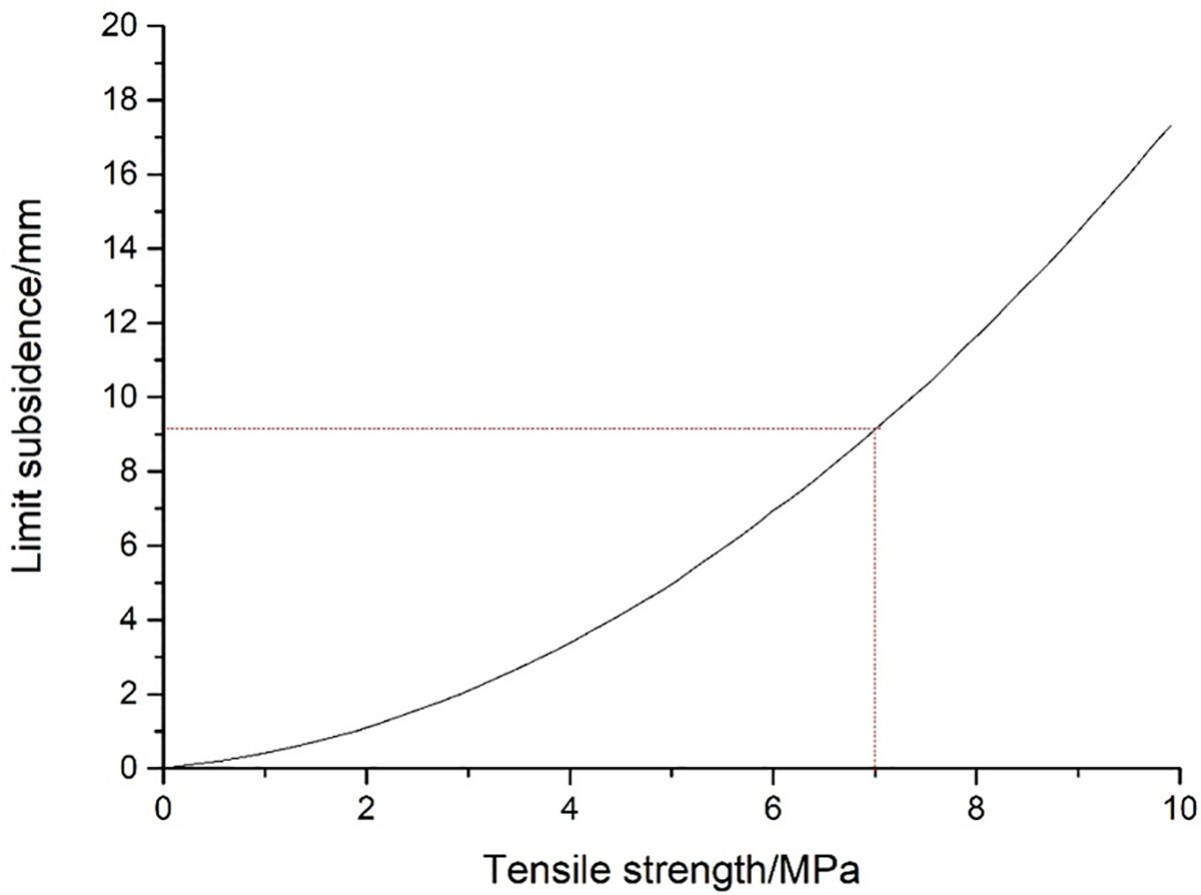
The relationship between maximum subsidence and tensile strength.

By substituting related data in Table 2 into Eq (7), the relationship between the maximum subsidence and the tensile strength of KS can be known, as shown in Fig 5. Because of small tensile strength of geological strata, the crack initiation takes place when the strata have just subsided for tens of millimeters.

**Table 2:** Occurrence state and mechanical parameters of prototype and model.

Serial number	Lithology	Prototype				Model			
		Slice thickness (m)	Cumulative mining depth(m)	Bulk density (kN/m3)	Compressive strength (MPa)	Slice thickness (cm)	Cumulative mining depth(cm)	Bulk density (kN/m3)	Compressive strength (MPa)
1	Floor	5	107.5	24	48	5	109	15	0.3
2	Coal	2.5	102.5	13	9	4	104	8.125	0.056
3	Immediate roof	4	100	22	40	4	100	1.4	0.25
4	KS1	2	96	27	76	2	96	16.9	0.475
5	Soft rock	12	94	22	40	12	94	13.75	0.25
6	KS2	4	82	27	76	4	82	16.875	0.475
7	Soft rock	23	78	22	40	23	78	13.75	0.25
8	KS3	5	55	27	76	5	55	16.875	0.475
9	Soft rock	35	50	22	40	35	50	13.75	0.25
10	PKS	10	15	27	76	10	15	16.875	0.475
11	Loose layer	5	5	15	20	5	5	9.375	0.125

In this paragraph, an example is given to calculate the limit subsidence at the moment of crack initiation of KS, and parameters required for the calculation are listed in Table 3. First, the load on KS is calculated according to Eq (3). Next, the position of KS can be determined according to the calculated load, hardness and stiffness, which suggests that No.1 and No. 5 strata are KS. Then, by substituting the load *q* = 0.35 MPa on No.1 stratum into Eq (7), *v*_*lim*_ = 9.1 mm can be obtained. After coal mining, the surface movement generally reaches a hundreds of millimeters, so KS in the continuous deformation zone have inevitably generated fractures. Besides, since the lithology of KS is stronger than other strata, it can be seen that all strata in the continuous deformation zone have fractured. Although the heterogeneity of force and the location of fixed ends are not taken into consideration when calculating the loading and subsidence of KS in the continuous deformation zone, it is foreseeable that they have little effect on the results. Therefore, the judgment that overburden starts to fracture under very slight subsidence is reasonable. It is worth noting that this only refers to cracks initiation of the KS, not both the macroscopic long fractures and break-through fractures of the KS.

**Table 3:** Physical and mechanical parameters of KS and soft rocks.

Number	Lithology	*γ*/KN/m^3^	*h*/m	*E*/MPa	*σ*_b_/MPa
5	Powder sand	25	11	23000	7
4	Mudstone	20	9	17000	2.2
3	Shale	22	7	18000	2
2	Mudstone	20	3	17000	2.2
1	Powder sand	25	10	23000	7

## Development form of overburden fractures and deformation and movement of strata of different fracture penetration ratios

### Development form of overburden fractures

**Fig 6:**
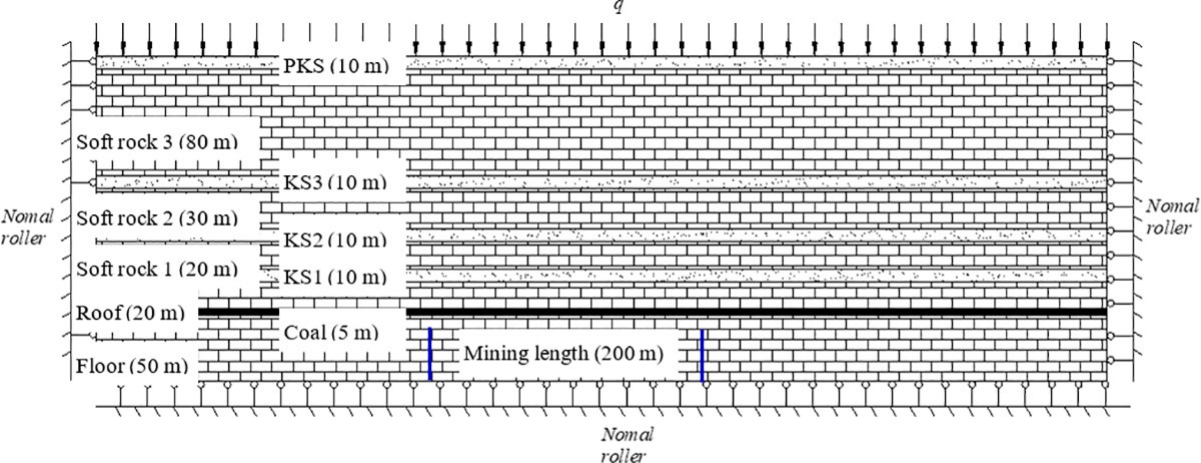
Schematic diagram of the fracture development in numerical simulation.

In this section, development form of fractures in overburden after coal mining was simulated using discrete element software (UDEC). The model was 400 m long and 205 m wide. The mining coal seam was 5 m thick, and each KS was 10 m thick. Located in the central of the model, the working face was 200 m wide. The x-direction displacement was fixed at its left and right boundaries; the y-direction displacement was fixed at its bottom boundary; and the loading was exerted from its upper boundary, as shown in Fig 6. The model adopted Mohr-Coulomb constitutive relations. The Physical and mechanical parameters of the rock mass and joints are shown in Tables 4 and 5.

**Table 4:** Physical and mechanical parameters of the rock mass used in UDEC.

Lithology	Height /m	Bulk modulus /GPa	Shear modulus /GPa	Cohesion /MPa	Friction /°	Tensile strength /MPa	Density /kg/m^3^
Floor	50	33.3	20	30	30	5	2000
Coal seam	5	7.5	3.46	10	20	2	1500
Roof	20	1.67	7.69	20	18	2	1800
KS1	10	27.8	20.8	40	25	80	2500
Soft rock	20	2.5	1.15	15	20	20	2000
KS2	10	33.3	25	40	35	95	2500
Soft rock	30	2.5	1.15	21	20	20	2000
KS3	10	8.5	6.15	40	35	50	2500
Soft rock	80	2.5	1.15	21	20	20	2000
PKS	10	33.3	25	40	35	50	2500

**Table 5:** Mechanical parameters of the joints used in numerical simulation.

Lithology	normal stiffness /GPa/m	shear stiffness /GPa	Cohesion /MPa	Friction /°	Tensile strength /MPa
Floor	73	5	4	21	0.5
Coal seam	3.3	4	0.3	8	00.0001
Roof	6	2	0.01	3	0.002
KS1	2	5	0.04	8	0.5
Soft rock	4.1	2	0.01	7	0.002
KS2	3	5	0.04	12	0.5
Soft rock	4.1	2	0.01	7	0.002
KS3	8.5	6	0.05	30	1
Soft rock	2.5	1.1	0.02	10	0
PKS	33	8	0.4	35	2

**Fig 7:**
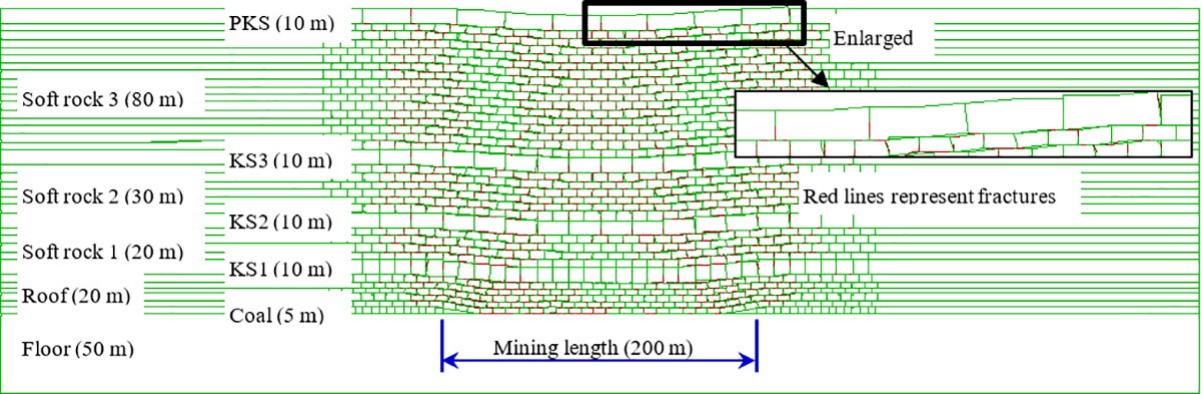
Fractures in the overburden after coal excavation in numerical simulation.

In the setting of model material parameters, the same parameter values of block were given to joint fractures. In this way, the generation of cracks in the rock mass can be determined by the appearance of identifiable joint fractures in the overburden after excavation. However, as the block can deform infinitely, it will not generate cracks even if stress exceeds the limit strength. Moreover, crack initiation and development in strata are different in appearance. Due to their small size, the initiated cracks cannot be identified in the model. Thus, numerical simulation cannot quantitatively calculate the relationship between crack initiation and subsidence, but just qualitatively determine whether KS in the continuous deformation zone have fractured. According to the formula of the height of water-conducting fractured zone (see Table 1), it can be obtained that KS3 and the strata above it are all within the range of continuous deformation zone. In the simulation results, fractures of different development degrees appear in KS3 and KS4, as shown in Fig 7, indicating that the continuous deformation zone has indeed undergone failure and generated cracks.

### Deformation and movement of strata of different fracture penetration ratios

In reality, when coal seam is mined to a certain extent, the surface will subside for at least hundreds of millimeters. In this case, theoretical analysis demonstrates that the whole overburden must have cracked. Thus, whether the method of continuum mechanics is applicable to the analysis of movement and deformation of strata in the continuous deformation zone still needs to be studied. This section provides some analysis using the numerical simulation.

In order to simulate the effect of fracture penetration ratio on the subsidence and deformation of overburden KS, finite element software (FLAC3D) was employed because the joints of discrete element software (UDEC) have great influence on the discrete element block sampling data, resulting in large fluctuations of horizontal movement. The size of the finite element model is consistent with that of the discrete element model (see Fig 7). The Physical and mechanical parameters of the rock mass are shown in Table 6.

**Table 6:** Physical and mechanical parameters of the rock mass used in FLAC3D.

Lithology	Height /m	Bulk modulus /GPa	Shear modulus /GPa	Cohesion /MPa	Friction /°	Tensile strength /MPa	Density /kg/m^3^
Floor	10	3.6	2.9	1.5	31	1.5	2400
Coal seam	4	2.6	1.9	1.2	21	0.5	1600
Roof	20	3	2.1	1.1	21	1.1	2100
KS1	5	6.6	4.9	3.2	31	3.5	2600
Soft rock	60	2	1.65	1.2	20	1	2100
PKS	10	30	26.5	8.2	38	10	2600

For strata with different thickness, the length of fractures may be different, so the simulation results are also different. Therefore, the simulation results of strata with different fracture length are not universal. In order to make the simulation results not affected by the thickness of strata and fracture length, a dimensionless concept, namely fracture penetration ratio, needs to be proposed. The fracture penetration ratio refers to the ratio of fracture length to the strata thickness in the expected direction of fracture development. Taking *δ* as the fracture penetration ratio, *h* as the strata thickness, *l* as the fracture length, and *α* as the angle between fracture and strata normal, then Eq (8) exists:

$$\delta =\frac{l\cos\alpha }{h}$$ (8)

**Fig 8:**
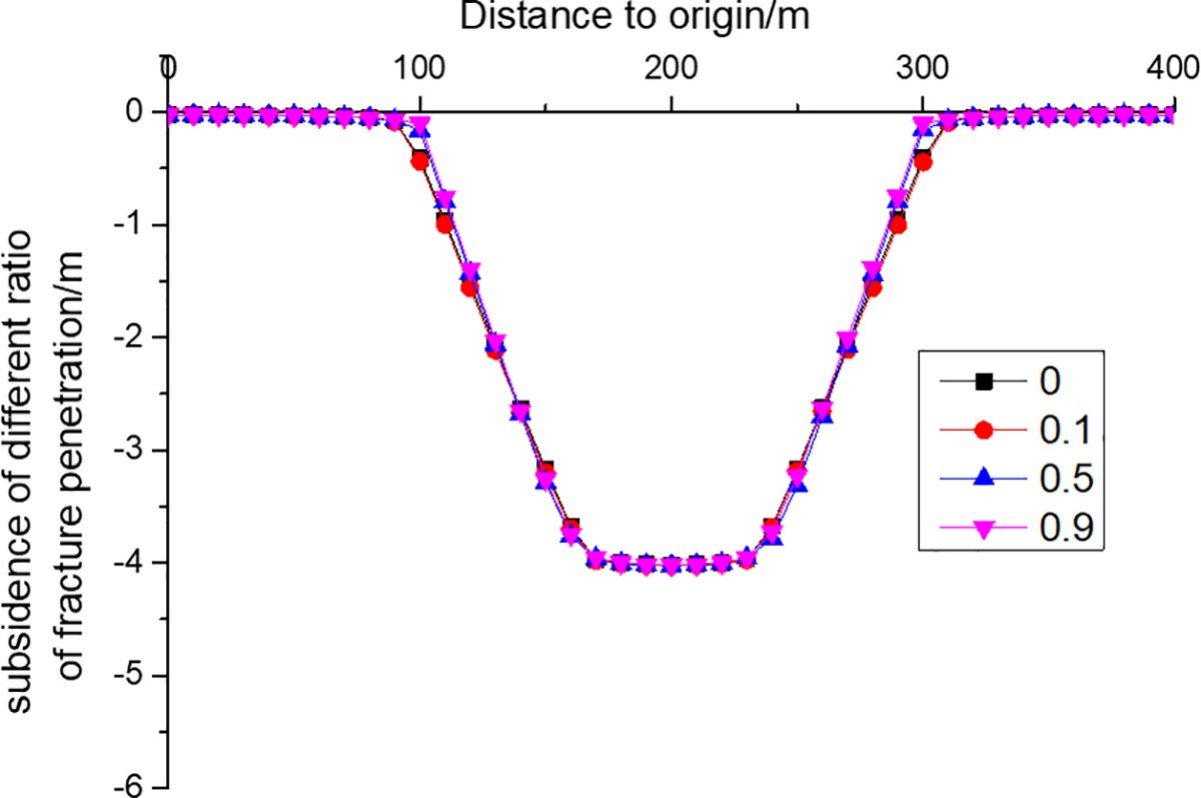
Subsidence of KS4 of different fracture penetration ratios.

In the finite element model, fractures were set in KS above the mining boundary, with one fracture on each side. Located at 100 m and 300 m in the horizontal direction, respectively, the fractures were perpendicular to the strata. Here, to reduce the interference factors of simulation and the workload of early model production, cracks are only set on the PKS. A total of four simulation schemes were implemented, namely, penetration ratios of 0, 0.1, 0.5 and 0.9, respectively. In the four schemes with different penetration ratios, the maximum subsidence of KS is the same, but the influence range of subsidence decreases slightly with the increase of penetration, as shown in Fig 8. This is because the loading causes the internal force to exceed the critical value of rock mass plastic failure. Before the strata below KS contact the bottom plate, the supporting force cannot limit the subsidence of KS; only the fractures weaken the transmission of horizontal force. The results show that the development of fractures has no significant effect on the subsidence of KS. Hence, the continuum mechanics method can be used to analyze the subsidence of KS, which provides a theoretical basis for applying continuum mechanics to the calculation of subsidence of strata that have fractured but have not broken yet within the overburden.

**Fig 9:**
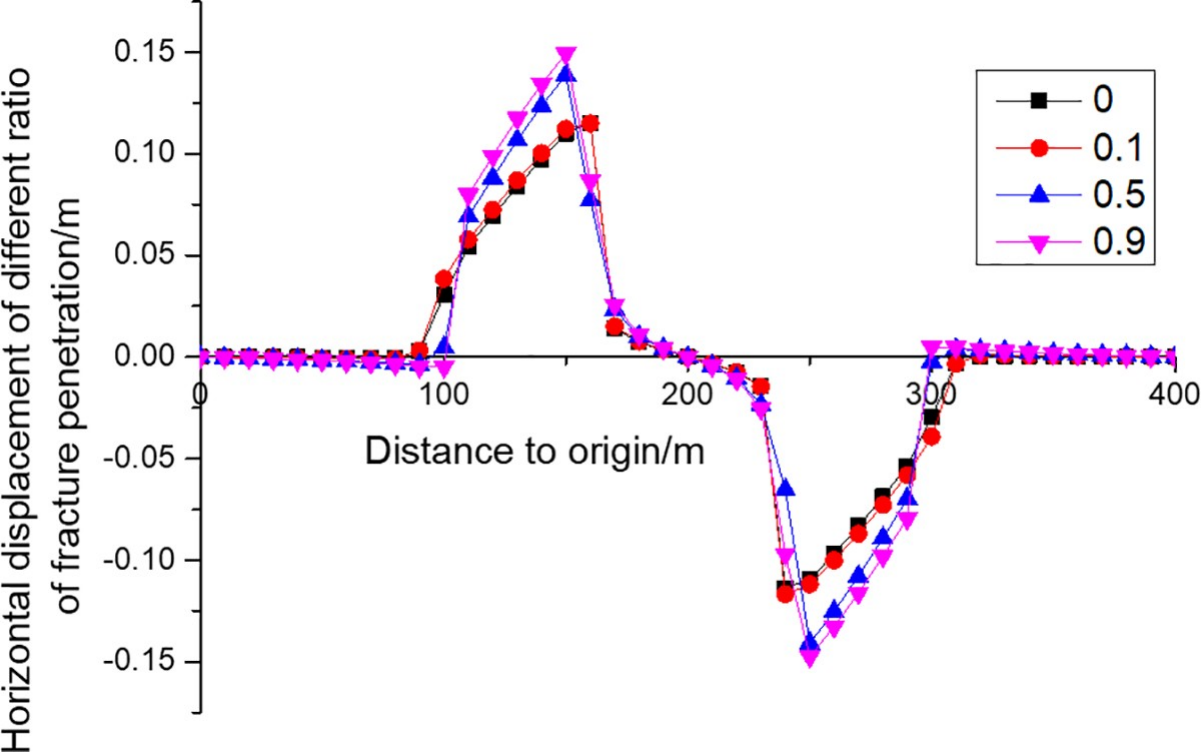
Horizontal displacements of KS4 of different fracture penetration ratios.

**Fig 10:**
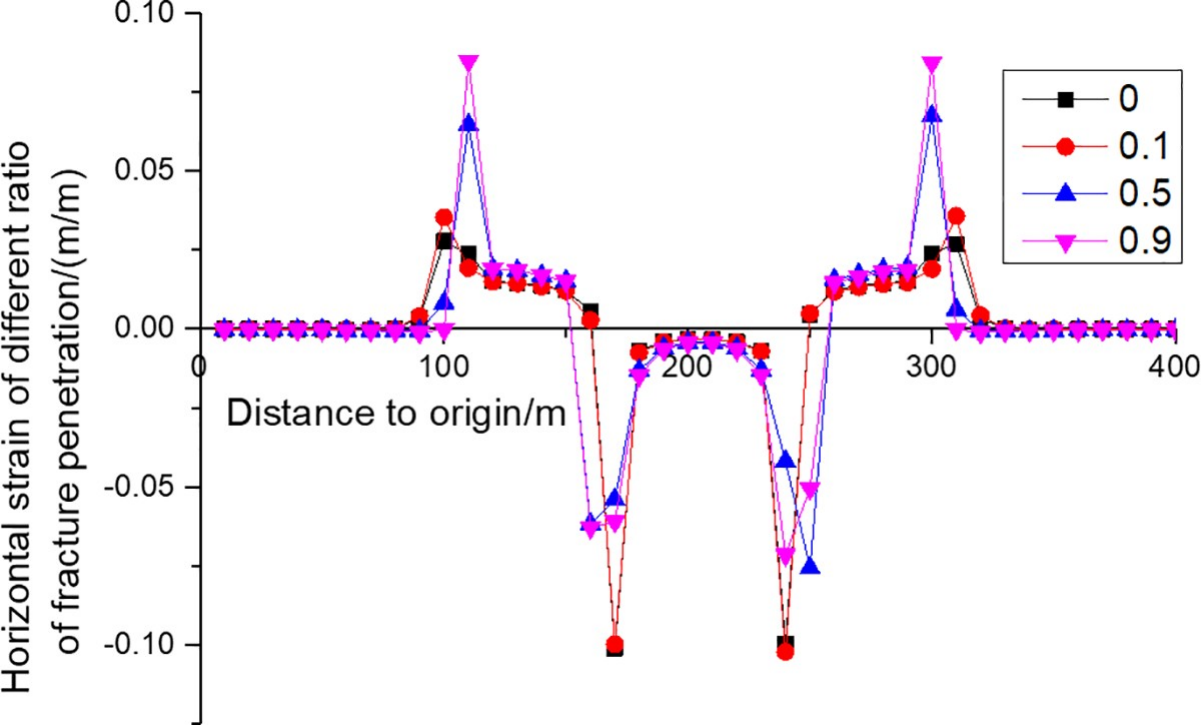
Horizontal strains of KS4 of different fracture penetration ratios.

Horizontal movement and horizontal deformation are important indicators of surface building evaluation [17]. The horizontal movement of KS is shown in Fig 9, from which it can be seen that fractures have two effects on the horizontal deformation of KS. One is that the horizontal movement range of KS gradually decreases with the increase of fracture penetration ratio, because the fractures weaken the transmission of horizontal force and thus reduce the transmission of horizontal displacement. The other is that the maximum horizontal movement value grows, because the increase of fracture penetration weakens the ability of coal side KS to limit the horizontal movement of KS above the goaf. The horizontal deformation of KS is shown in Fig 10, from which it can be seen that fractures have three effects on the horizontal deformation of KS. The first is that the range of horizontal deformation is reduced as the fracture penetration ratio increases, indicating that buildings beyond a certain range can be protected by digging ditches or grooves. The second is that the maximum tensile deformation increases, suggesting that buildings within the tensile range are more likely to be damaged as the fracture penetration increases. The third is that the maximum compression deformation falls, showing that buildings within the compression range are less likely to undergo compressed failure as the fracture penetration increases.

## Conclusions

1. The height of fracture development is the height of entire overburden if the surface subsides several centimeters. Strata start to fracture when they have subsided for just a few millimeters. In actual mining, the surface subsidence usually reaches a few meters, so all the strata must have fractured when the coal seam is mined to a certain extent. As a result, fractures of various development degrees are distributed in the whole overburden if the surface subsides several centimeters.
2. The subsidence of strata which have fractured but have not broken yet can be calculated through the continuum mechanics method. The fracture has almost no effect on the subsidence of KS. Therefore, no matter whether there are fractures in KS, the continuum mechanics method can be used to calculate the subsidence of KS, so as to predict the subsidence of surface. However, with the increase of fracture penetration ratio, the surface deformation range and the maximum compression deformation decrease, while the maximum horizontal movement and maximum horizontal tensile deformation increase, so large errors may exist when continuous mechanics is used to calculate the deformation.
